# Genetic Determinants of the Pharmacokinetic Variability of Rifampin in Malawian Adults with Pulmonary Tuberculosis

**DOI:** 10.1128/AAC.00210-17

**Published:** 2017-06-27

**Authors:** Derek J. Sloan, Andrew D. McCallum, Alessandro Schipani, Deirdre Egan, Henry C. Mwandumba, Steve A. Ward, David Waterhouse, Gertrude Banda, Theresa J. Allain, Andrew Owen, Saye H. Khoo, Geraint R. Davies

**Affiliations:** a Malawi Liverpool Wellcome Trust Clinical Research Programme, College of Medicine, University of Malawi, Blantyre, Malawi; b School of Medicine, University of St. Andrews, North Haugh, St. Andrews, Fife, United Kingdom; c Liverpool School of Tropical Medicine, Liverpool, United Kingdom; d Department of Medicine, College of Medicine, University of Medicine, Blantyre, Malawi; e Royal Liverpool University Hospital, Liverpool, United Kingdom; f Institute of Translational Medicine, University of Liverpool, Liverpool, United Kingdom; g Institute of Infection and Global Health, University of Liverpool, Liverpool, United Kingdom

**Keywords:** tuberculosis, pharmacokinetics, single nucleotide polymorphism, SLCO1B1, AADAC, CES-1

## Abstract

Variable exposure to antituberculosis (TB) drugs, partially driven by genetic factors, may be associated with poor clinical outcomes. Previous studies have suggested an influence of the *SLCO1B1* locus on the plasma area under the concentration-time curve (AUC) of rifampin. We evaluated the contribution of single nucleotide polymorphisms (SNPs) in *SLCO1B1* and other candidate genes (*AADAC* and *CES-1*) to interindividual pharmacokinetic variability in Malawi. A total of 174 adults with pulmonary TB underwent sampling of plasma rifampin concentrations at 2 and 6 h postdose. Data from a prior cohort of 47 intensively sampled, similar patients from the same setting were available to support population pharmacokinetic model development in NONMEM v7.2, using a two-stage strategy to improve information during the absorption phase. In contrast to recent studies in South Africa and Uganda, SNPs in *SLCO1B1* did not explain variability in AUC_0–∞_ of rifampin. No pharmacokinetic associations were identified with *AADAC* or *CES-1* SNPs, which were rare in the Malawian population. Pharmacogenetic determinants of rifampin exposure may vary between African populations. *SLCO1B1* and other novel candidate genes, as well as nongenetic sources of interindividual variability, should be further explored in geographically diverse, adequately powered cohorts.

## INTRODUCTION

Although effective chemotherapy for tuberculosis (TB) has been available for several decades, cure rates are variable, remaining at 34 to 76% in many countries (1–6). The traditional view that treatment failure, relapse, and emergence of antimicrobial resistance are predominantly driven by poor adherence has been challenged by reports that interindividual variability in pharmacokinetic (PK) exposure to rifampin (RIF) accounts for some unfavorable outcomes, even among patients who do not miss doses of medication (7).

Existing clinical data show up to 10-fold interindividual variability in the plasma PK indices of key drugs, especially RIF (8–11). Maximum plasma concentration (*C*_max_) and area under the concentration-time curve (AUC) measurements are often reported as “low,” suggesting that some patients are underdosed according to currently clinical recommendations. Patient physiology, comorbidities, concomitant medications, and dietary intake all influence drug exposure. However, genetic polymorphisms in drug metabolizing enzymes and transporters may explain up to 30% of the PK variability for all drugs (12–14). Although Africans have the highest degree of genetic diversity worldwide (15) and sub-Saharan Africa accounts for a large proportion of global TB incidence and mortality, data on the pharmacogenetic determinants of antituberculosis drug exposure among TB-endemic African populations are sparse.

RIF is believed to be the decisive drug that enables short-course chemotherapy. Metabolism by hepatic esterases and biliary excretion of RIF occurs after first-pass metabolism and hepatocellular uptake, which may be primarily mediated by organic anion-transporting polypeptide 1B1 (OATP1B1), the product of the gene *SLCO1B1* (16, 17). Reports from South Africa and Uganda suggested that two *SLCO1B1* single nucleotide polymorphisms (SNPs), rs11045819 and rs4149032, are common in African patients and associated with reduced RIF plasma exposure (18–20). However, a recent study from a TB-endemic population in southern India reported lower variant allele frequency of these SNPs than the prior African work and no genotypic effect on the pharmacokinetics of RIF (21). More data from a broader range of global settings are required to assess the effect of SLCO1B1 genetics on rifampin exposure.

Within the hepatocyte, RIF is thought to be metabolized by microsomal hepatic esterases, which have been incompletely characterized to date. *In vitro* data have suggested that the serine esterase arylacetamide deacetylase (*AADAC*) may mediate 25-deacetylation of rifamycins (22) and that SNPs in the genes encoding hepatic microsomal AADAC may alter RIF clearance (23). Alternative routes to breaking ester linkages in RIF metabolism may involve the carboxyesterase (e.g., CES-1 and -2) enzymes which are relatively abundant in the liver and gut. There are no clinical data to define the effect of SNPs in *AADAC* or *CES* genes on plasma RIF exposure.

To assess previously described pharmacogenetic effects in a new population, and evaluate the contribution of unexplored polymorphisms in other key metabolic processes, we assessed the impact of critical SNPs in the candidate genes *SLCO1B1*, *AADAC*, and *CES-1* on plasma exposure to RIF among Malawian adults with smear-positive pulmonary TB. To maximize our study size with limited resources in a low-income setting, participants underwent sparse PK sampling at two time points. Prior intensive PK data from the same population facilitated use of two-stage population PK methods to improve information on RIF exposure, particularly during the absorption phase.

## RESULTS

The sparsely sampled data set comprised 174 participants. A total of 121 (69.5%) were male, the median age was 30 years (range, 17 to 61 years), and the median weight was 52.0 kg (range, 34 to 74 kg). Of these participants, 98 (56.3%) were HIV infected, with a median CD4 count of 174 cells/μl (range, 6 to 783 cells/μl). Of 98 HIV-infected patients, 28 (28.6%) were on antiretroviral therapy (ART) at the time of recruitment. Full details are provided in Table 1.

**TABLE 1:** Demographic and clinical description of cohort

Characteristic^*a*^	Total (*n* = 174)
Age in yrs, median (range)	30 (17–61)
Male, *n* (%)	121 (69.5)
HIV infected, *n* (%)	98 (56.3)
CD4 in HIV-infected patients in cells/μl, median (range)	174 (6–783)
On ART at baseline if HIV infected, *n* (%)	28 (28.6)
Wt in kg, median (range)	52 (34–74)
Adherence, *n* (%)^*b*^	
Missed no doses	143 (82.2)
Missed <2 doses	10 (5.7)
Missed >2 doses	2 (1.1)
Rifampin/isoniazid dose in mg, *n* (%)	
300/150	2 (1.1)
450/225	113 (64.9)
600/300	59 (33.9)
*SLCO1B1* mutations	
rs11045819	
Wild (CC)	150 (86.2)
Heterozygote (AC)	24 (13.8)
Variant (AA)	0 (0)
rs4149032	
Wild (TT)	89 (51.1)
Heterozygote (CT)	59 (33.9)
Variant (CC)	26 (14.9)
*AADAC* mutations	
rs1803155	
Wild (GG)	3 (1.7)
Heterozygote (CG)	80 (46.0)
Variant (CC)	91 (52.3)
rs61733692	
Wild (TT)	174 (100)
Heterozygote (CT)	0 (0)
Variant (CC)	0 (0)
*AADAC* haplotype	
AADAC*1/*1	3 (1.7)
AADAC*1/*2	80 (46.0)
AADAC*2/*2	91 (52.3)
*CES1* mutations	
rs12149368	
Wild (GG)	173 (99.4)
Heterozygote (CG)	1 (0.6)
Variant (CC)	0 (0)

a ART, antiretroviral therapy; *SLCO1B1*, solute carrier organic anion transporter family member 1B1; *AADAC*, arylacetamide deacetylase; *CES1*, carboxylesterase 1. *n*, number of patients.

b Adherence data were only available for 155 patients.

The intensely sampled data set used for the first stage of pharmacokinetic model-fitting comprised 47 participants: 24 (52%) were male, the median age was 34 years (range, 16 to 60 years), and the median weight was 52.5 kg (range, 35.8 to 74.3 kg). Of the 47 participants, 30 (65%) were HIV infected and 13 (27.7%) were on ART at the time of recruitment. All participants from both cohorts were black Africans newly diagnosed with TB at Queen Elizabeth Central Hospital (QECH), Blantyre, Malawi.

In the first stage of the PK analysis, performed with the intensively sampled data set alone, a one-compartment model appeared most appropriate with a transit compartment model best describing the absorption phase (Δ objective function value [OFV] = 111). Interindividual random effects (IIV) were supported for the apparent clearance (CL/*F*), apparent volume of distribution (*V*/*F*), and absorption mean transit time (MMT).

The second stage of the analysis utilized only the sparsely sampled data set. Due to the relative lack of information on the absorption phase from this data set, model parameters relating to absorption were fixed at the values estimated in the first stage of the analysis. Model refinement involved a comprehensive search of the covariates available. The inclusion of an allometric scaling weight model decreased the OFV significantly (ΔOFV = 7). The only demographic covariate which significantly affected the model fit was sex, with a decrease in the OFV of 10.5 (*P* < 0.01). The final model showed an increase of clearance of 17% in male patients, resulting in a decreased of average AUC of 3% in males. Full PK parameters of the final two-stage RIF model are included in Tables 2 and 3.

**TABLE 2:** Parameter value estimates for the base model stage 1^*a*^

Parameter	Typical value	%RSE	95% CI
CL/F (liters/h)	19.5	3.9	18.2–20.8
*V*/F (liters)	27.1	13.9	20.9–33.3
*K_a_* (h^−1^)	0.277	9.8	0.23–0.32
NN	1.5	41	0.09–3.9
MMT (h)	0.326	35	0.05–1.0
Random effects			
IIVCL	0.165	15.3	0.12–0.20
IIVV	0.425	27.1	0.25–0.59
IIVMMT	0.0706	75	0.01–1.79
Residual variability			
Proportional error (%)	0.23	7.8	0.20–0.26

a CL/F, clearance (F is unknown bioavailability); *V*/F apparent volume of distribution; *K_a_*, absorption rate constant; NN, number of transit compartment; MMT, absorption mean transit time; IIVCL, interindividual variability on clearance; IIVV, interindividual variability on volume; IIVMMT, interindividual variability on absorption mean transit time. RSE, relative standard error reported. CI, confidence interval.

**TABLE 3:** Parameter value estimates for the final model stage 2^*a*^

Parameter	Typical value (shrinkage [%])	%RSE	95% CI
CL/F (liters/h)	19.6	12	16.7–22.5
*V*/F (liters)	23.6	9	17.1–30.1
*K_a_* (h^−1^)	0.277 (FIX)		
NN	1.5 (FIX)		
MMT (h)	0.326 (FIX)		
THETA_sex^male^	1.2	13	1.0–1.3
Random effects			
IIVCL	0.076 (22)	29	0.033–0.11
IIVV	0.397 (28)	29	0.17–0.63
IIVMMT	0.0706 (FIX)		
Residual variability			
Proportional error (%)	0.22	12	0.19–0.26

a CL/F, clearance (F is unknown bioavailability); *V*/F apparent volume of distribution; FIX, value was fixed after model stage 1; NN, number of transit compartment; MMT, absorption mean transit time; THETA_sex^male^, fractional change in clearance for males; IIVCL, interindividual variability on clearance; IIVV, interindividual variability on volume; IIVMMT, interindividual variability on absorption mean transit time. RSE, relative standard error reported. CI, confidence interval.

**FIG 1:**
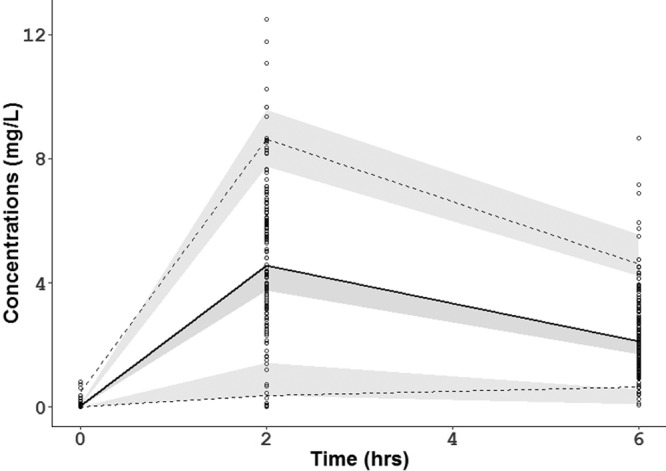
Visual predictive check for the final rifampin model. The lower, middle, and upper lines are the 5th percentile, the median, and 95th percentile for the observed data, respectively. The shaded areas are the 95% confidence intervals for the 5th percentile, the median, and the 95th percentile for the simulated data.

A visual predictive check (VPC) of 1,000 simulated data sets indicated that the final model performed adequately (Fig. 1). The values of RIF AUC_0–∞_ and *C*_max_ were obtained using empirical Bayesian estimates of the individual parameters. A predicted RIF AUC_0–∞_ was generated for each subject using their estimated CL/*F* from the final model (dose/[CL/*F*] = AUC). The median predicted AUC_0–∞_ was 29.9 μg·h/ml, with a range of 19.7 to 63.4 μg·h/ml. The median *C*_max_ was 4.8 μg/ml, with a range of 1.4 to 10.9 μg/ml.

Candidate SNPs in the *SLCO1B1*, *AADAC*, and *CES-1* loci were assessed. The distribution of alleles at these loci is presented in Table 1. The rs11045819 *SLCO1B1* SNP was rare, existing at an overall frequency of 0.07, while the rs4149032 *SLCO1B1* SNP had a minor allele frequency of 0.32. No subjects possessed the variant rs61733692 *AADAC* SNP, while the rs1803155 *AADAC* SNP had a minor allele frequency of 0.25. Only one patient was heterozygote for the variant rs121493368 *CES-1* SNP. Since the metabolic action of these genes is believed to be exerted largely during first-pass metabolism, their impact on relative bioavailability (*F*) was tested, as well as on CL/*F*. Additive, dominant, and recessive models of effect were tested for each SNP. The inclusion of *SLCO1B1* genotypes did not significantly improve the model fit. The *AADAC* SNPs and haplotype, or the *CES-1* SNP, did not significantly alter the RIF model fit, as would be anticipated from the very low variant allele frequency in our study population.

## DISCUSSION

This study is the first pharmacogenetic analysis of antituberculosis therapy from Malawi, the largest such study to date using a population modeling approach to relate pharmacogenetic polymorphisms to plasma AUC and the first to evaluate the effect of SNPs in *AADAC* in this context. The results of our analysis draw attention to the importance of (i) fully considering SNP diversity within Africa, (ii) the complexity of competing metabolic processes, and (iii) recognizing the limitations of observational pharmacogenetic designs.

A two-stage population PK modeling approach was used to quantify RIF exposure among adult patients on treatment for pulmonary TB, benefiting from the availability of data from a preexisting, exchangeable population of intensively sampled patients (24). A sequential rather than simultaneous approach was used to handle all the data because pharmacogenetic information was unavailable for the intensive data set, reducing the value of combined modeling with incomplete covariates.

The two-step approach enabled plausible and efficient estimation of plasma AUC from sparsely sampled participants, which is a more reliable measure of total drug exposure than a single “peak” concentration at a fixed time after dosing. Plasma AUC demonstrated up to 6-fold variability between patients, like previous African cohorts. RIF is a crucial component of the current first-line anti-tuberculosis regimen. In general, AUC/MIC (MIC) is the PK index most closely related to the bactericidal activity of RIF (25), and a recent South African study reported a threshold of AUC of RIF (≤13 μg·h/ml) to be an independent predictor of poor outcome (26). Therefore, identifying host pharmacogenetic factors that influence the AUC of this key drug may help to identify individuals at risk of treatment failure and explain differences in treatment outcomes between populations.

The *SLCO1B1* locus encodes an organic anion transporter (OATP1B1) implicated in the hepatic uptake of several drugs and contains at least 17 nonsynonymous SNPs. Much interest in Europe has focused on the role of rs4149056 (521T>C, *5) in toxicity of statin therapy (27), while studies in South Africa and Uganda have implicated two *SLCO1B1* SNPs (rs4149032 and rs11045819) in reduced RIF exposure (18–20). However, in keeping with a recent report from southern India (21), inclusion of these genotypes did not significantly improve the PK model fit for RIF in our study.

There are potential explanations for the discrepant results between *SLCO1B1* studies. The rs4149032 variant allele frequency was higher in South Africa (0.70 to 0.76), where an association with RIF was reported, than our Malawian study (0.32) or southern India (0.46) where the results were negative. Similarly, the rs11045819 variant allele frequency was higher in Uganda (0.15) that Malawi (0.07) or India (0.01). Therefore, it is possible that populations with lower allele frequencies require a larger sample size for adequate power to detect a pharmacokinetic effect. In addition, while rs11045819 is believed to be functional (463C>A, *4), rs4149032 is an intronic SNP of unclear functional status which, in European populations, is in strong linkage disequilibrium with rs11045819 (28). However, these SNPs had a low level of linkage disequilibrium (D′ 0.16) in Malawi, echoing findings from South Africa (18) and southern India (21). There are large differences in linkage disequilibrium worldwide and within Africa (15), so it is possible that previously recognized SNPs may tag functional genes in some populations but not others.

AADAC, a microsomal serine deacetylase expressed mainly in the liver and gastrointestinal tract, is responsible for 25-deacetylation of rifamycins *in vitro* (23). Previous preclinical studies identified that expression of the AADAC*3 allele (rs1803155/rs61733692) significantly reduced RIF clearance (22). No subjects in our study possessed the AADAC*3 allele, and inclusion of *AADAC* haplotype did not significantly improve the RIF model fit. Although the low incidence of these *AADAC* SNPs excluded their role in explaining interindividual PK variability in this Malawian population, these genes are understudied. Similarly, the low incidence of one *CES-1* SNP in our population does not exclude the possibility that polymorphisms in this gene are relevant in other populations or that other mutations are important. Future work may describe higher SNP incidence in other settings, or identify alternative SNPs of relevance to drug exposure.

The gene loci assessed here focused on hepatic drug uptake and metabolism. Additional processes, including widely distributed mucosal P-glycoprotein transporters (29) and orphan nuclear receptor regulatory elements which influence gene induction (30) may also influence RIF metabolism. Investigation of these broader pharmacogenetic factors may be studied separately.

These data indicate that interindividual variability in RIF PK among Malawian adults was not explained by polymorphisms in the candidate genes studied. They illustrate the importance of local ethnic background in the context of high SNP diversity within Africa and emphasize the need for caution in extrapolating findings across the continent. Growing recognition of the extent and clinical consequences of variable RIF PK (31–37), coupled with recent evidence that dose escalation is safe and tolerable (38), highlights the need for ongoing work to better define the relative importance of polymorphisms and nongenetic risk factors for low antibiotic exposure in a range of populations.

There were several limitations to our study. Standard fixed-dose-combination (FDC) tablets were used in treatment of the participants under field conditions, but we have not been able to account for the effect of quality of the potentially varying drug formulation on PK variability between the two cohorts. This reflects the reality of the TB drug supply chain in most countries. The use of sparse PK sampling at three fixed time points over 6 h limited the precision of the estimates in each model, particularly for the absorption parameters. The use of data from a similar, intensively sampled cohort to develop a population PK model mitigated this to some extent, achieving reasonable precision for the key PK parameter, i.e., AUC. Finally, the size of the data set was not predicated on this secondary pharmacogenetic analysis, and the negative findings could be due to a relative lack of power. However, our study was larger than any reported African cohort to date, and there are limitations on the number of PK profiles, even based on sparse sampling protocols, that can practically be obtained in the field situation. On the other hand, it is widely recognized that candidate gene studies can be prone to chance findings which may not be subsequently replicated.

In conclusion, the high interindividual variability in plasma exposure to RIF among Malawian adults with pulmonary TB cannot be explained by genetic heterogeneity in *SLCO1B1*, as suggested from other African populations. Similarly, the variability cannot be explained by the novel candidate gene SNPs in *AADAC* and *CES-1* that were evaluated, but the low frequency of variant genes in our population does not exclude an association elsewhere. The true significance of pharmacogenetic influences on disposition of antituberculosis drugs compared to nongenetic factors may only be established through consistent observation across geographically diverse and adequately powered cohort studies.

### Ethics.

Ethical approval was obtained from the College of Medicine Research Ethics Committee, University of Malawi, and the Research Ethics Committee of Liverpool School of Tropical Medicine.

## MATERIALS AND METHODS

### Study participants.

The sparse pharmacokinetic data sampling was conducted within a prospective cohort study conducted at QECH in Blantyre, Malawi, from 2010 to 2012. Consenting adults aged 16 to 65 years with sputum smear-positive pulmonary TB were eligible. Exclusion criteria included a hemoglobin level of <6 g/dl, a creatinine level of >177 μmol/liter, a total bilirubin level of >51 μmol/liter, an alanine transaminase level of >200 IU/liter, a clinical status suggestive of imminent mortality (World Health Organization [WHO] performance score of 4 [39]), pregnancy, TB treatment within 5 years, corticosteroid therapy, or baseline resistance to rifampin and isoniazid determined using a GenoType MTBDRplus 2.0 line probe assay (Hain Life Sciences). Patient characteristics have been reported previously (40). Participants received daily FDC tablets according to a WHO-approved weight-adjusted regimen (including rifampin at 8 to 12 mg/kg) and standard National Tuberculosis Programme guidelines (41). Adherence was monitored by direct questioning and pill counts. All patients had point-of-care HIV serology. ART was provided in accordance with national protocols.

The intensively sampled pharmacokinetic data used for the first stage of model construction was obtained from a prior study of adult patients with sputum smear-positive pulmonary TB at the same hospital, conducted from 2007 to 2008. The recruitment criteria, patient characteristics, and treatment protocols have also been reported previously (24) and were similar to the sparse sampling data set.

### Genotyping.

Genomic DNA extraction and genotyping were performed from whole blood samples collected at baseline from each patient, as previously described (18). TaqMan real-time PCR using fluorescent probes for allelic discrimination was used to detect SNPs of two RIF-transporting or -metabolizing genes: *SLCO1B1* (rs11045819 and rs4149032) and *AADAC* (rs1803155 and rs61733693). These SNPs are previously reported to have functional significance for RIF PK (18, 22, 23). An exploratory SNP (rs12149368) in *CES-1* was also assessed.

### Drug plasma concentration determination.

For the sparse sampling cohort, blood collections to measure steady-state RIF concentrations were undertaken on day 14 or 21 after TB treatment initiation. Patients attended the study clinic at 7:30 a.m. after an overnight fast. Samples were collected predose and then again 2 and 6 h after administration of the medications. Plasma was separated by centrifugation and stored at −70°C until analysis.

RIF concentrations were determined using a liquid chromatographic/tandem mass spectrometry method (24) using appropriate internal standards validated to internationally recognized acceptance criteria as previously described (42). The lower limit of quantification (LLQ) was 0.5 μg/ml for RIF. A total of 5% of the samples had RIF results below the LLQ. These data points were handled by imputing a value which was 50% of the LLQ.

### Population pharmacokinetic analysis.

A population PK model for RIF was developed using NONMEM (version 7.2.0; ICON Development Solutions). Since the sparse sampling data contained little information on RIF absorption, a two-stage model-building strategy was used.

In the first stage, a data set previously obtained from 47 adult tuberculosis patients in Blantyre employing intensive sampling, and the same drug assay (24) was used to characterize the absorption phase. No covariates were included. One- and two-compartment models with alternative models of absorption were fitted to the data using the first-order conditional method of estimation with interaction. Among the models explored were simple first-order absorption or a sequence of zero- and first-order absorption incorporating either lag times or transit compartment absorption. Proportional, additive, and combined proportional and additive error models were considered to describe residual variability. The minimal OFV (equal to −2 log likelihood) was used as a goodness-of-fit metric, with a decrease of 3.84 corresponding to a statistically significant difference between models (*P* = 0.05, χ^2^ distribution, one degree of freedom). Residual plots were also examined.

Once the appropriate structural model was established, the values of the absorption parameters were fixed, and a second-stage analysis was performed using data from the sparsely sampled participants of the present study. The following covariates were explored: body weight, age, gender, HIV status, and SNP genotypes.

Exponential errors following a log-normal distribution were assumed for the description of interindividual variability in pharmacokinetic parameters, as shown in the following equation:

(1) $$\theta_{xi}=\theta_{x}\times \exp\left(\eta_{xi}\right)$$

where θ*_xi_* is pharmacokinetic parameter “*x*” of the *i*th individual, θ_*x*_ is the population parameter estimate, and η*_xi_* is the log interindividual variability for parameter “*x*” drawn from a normal distribution with a mean of zero and variance ω^2^. RIF was administered orally, CL and *V* represent apparent values (CL/*F* and *V*/*F*, respectively, where *F* is the oral bioavailability, which was fixed to 1). An allometric weight model was applied to standardize the pharmacokinetic parameters using a standard weight (wt_std_) of 70 kg. An allometric weight model for clearance parameters is given by CL/*F*_wt_ = (wt/wt_std_)^3/4^ and for volume parameters is given by *V*/*F*_wt_ = (wt/wt_std_)^1^, where CL/*F*_wt_ and *V*_wt_ are the weight functions for the clearance parameters and the volume of distribution parameters, respectively, and wt is the individual weight value.

Dichotomous covariates were introduced as a power model and continuous variables were modeled using a power model with normalized covariate:

(2) $$\theta_{i}=\theta_{1}\times \theta_{{\text{COV}}^{x}}$$

(3) $$\theta_{i}=\theta_{1}\times {\left({\text{COV}}_{i}/{\text{COV}}_{\text{median}}\right)}^{\theta_{\text{COV}}}$$

where θ*_i_* is the pharmacokinetic parameter of the *i*th individual and θ_1_ is the population parameter estimate. In equation 2 (dichotomous covariates), θ_cov_ is the ratio value of θ*_i_* for the individuals *x* = 0 or 1. In equation 3 (continuous covariates), COV_*i*_ is the value of the covariate for the *i*th individual, COV_median_ is the median value, and θ_cov_ is the fraction of θ*_i_* relative to the considered covariate effect.

Genotype information was coded as an index variable, shown below for CL/*F*: TVCL = θ_0_ + θ_1_·*X*_1_ + θ_2_·*X*_2_, where θ_0_ is the typical value of CL/*F* for individuals with homozygosity, θ_1_ is the relative difference in CL/*F* for heterozygous for the mutant allele when *X*_1_ = 1, and θ_2_ is the relative difference in CL/*F* for patients homozygous for the mutant allele when *X*_2_ = 1.

Graphical methods were used to explore the relationship of covariates versus individual predicted pharmacokinetic parameters. Each covariate was introduced separately into the model and only retained if inclusion in the model produced a statistically significant decrease in the OFV of 3.84 (*P* ≤ 0.05). A backward elimination step was then carried out once all relevant covariates were incorporated, and covariates were retained if their removal from the model produced a significant increase in the OFV (>6.63 points; *P* ≤ 0.01, χ^2^ distribution, one degree of freedom).

To perform a VPC using Perl-speaks-NONMEM (PsN), 1,000 data sets were simulated using the parameter estimates defined by the final model with the SIMULATION SUBPROBLEMS option of NONMEM. From the simulated data, 90% prediction intervals (P5 of P95) were constructed. Observed data from the original data set were superimposed for both regimens. PsN was used to run a nonparametric bootstrap of 200 iterations to provide unbiased estimates of the standard errors and the 95% confidence intervals of the estimated parameters. Estimates of AUC_0–∞_ for RIF were calculated from simulated values of CL/*F* by using the following equation: AUC_0–∞_ = [(dose ∈ milligrams)/(CL/*F*)].

## References

[1] Abdool Karim SS, Naidoo K, Grobler A, Padayatchi N, Baxter C, Gray A, Gengiah T, Nair G, Bamber S, Singh A, Khan M, Pienaar J, El-Sadr W, Friedland G, Abdool Karim Q 2010 Timing of initiation of antiretroviral drugs during tuberculosis therapy. N Engl J Med 362:697–706. doi:10.1056/NEJMoa0905848. 20181971 PMC3076221

[2] Karumbi J, Garner P 2015 Directly observed therapy for treating tuberculosis. Cochrane Database Syst Rev 200:CD003343. doi:10.1002/14651858.CD003343.pub4. PMC4460720 26022367

[3] Visser ME, Grewal HM, Swart EC, Dhansay MA, Walzl G, Swanevelder S, Lombard C, Maartens G 2011 The effect of vitamin A and zinc supplementation on treatment outcomes in pulmonary tuberculosis: a randomized controlled trial. Am J Clin Nutr 93:93–100. doi:10.3945/ajcn.110.001784. 21068353

[4] World Health Organization. 2016 Global tuberculosis report. World Health Organization, Geneva, Switzerland http://www.who.int/tb/publications/global_report/en/.

[5] Hesseling AC, Walzl G, Enarson DA, Carroll NM, Duncan K, Lukey PT, Lombard C, Donald PR, Lawrence KA, Gie RP, van Helden PD, Beyers N 2010 Baseline sputum time to detection predicts month two culture conversion and relapse in non-HIV-infected patients. Int J Tuberc Lung Dis 14:560–570. 20392348

[6] MacKenzie WR, Heilig CM, Bozeman L, Johnson JL, Muzanye G, Dunbar D, Jost KC Jr, Diem L, Metchock B, Eisenach K, Dorman S, Goldberg S 2011 Geographic differences in time to culture conversion in liquid media: Tuberculosis Trials Consortium study 28. Culture conversion is delayed in Africa. PLoS One 6:e18358. doi:10.1371/journal.pone.0018358. 21494548 PMC3073969

[7] Srivastava S, Pasipanodya JG, Meek C, Leff R, Gumbo T 2011 Multidrug-resistant tuberculosis not due to noncompliance but to between-patient pharmacokinetic variability. J Infect Dis 204:1951–1959. doi:10.1093/infdis/jir658. 22021624 PMC3209814

[8] Wilkins JJ, Langdon G, McIlleron H, Pillai GC, Smith PJ, Simonsson US 2006 Variability in the population pharmacokinetics of pyrazinamide in South African tuberculosis patients. Eur J Clin Pharmacol 62:727–735. doi:10.1007/s00228-006-0141-z. 16685561

[9] Jonsson S, Davidse A, Wilkins J, Van der Walt JS, Simonsson US, Karlsson MO, Smith P, McIlleron H 2011 Population pharmacokinetics of ethambutol in South African tuberculosis patients. Antimicrob Agents Chemother 55:4230–4237. doi:10.1128/AAC.00274-11. 21690284 PMC3165318

[10] Wilkins JJ, Langdon G, McIlleron H, Pillai G, Smith PJ, Simonsson US 2011 Variability in the population pharmacokinetics of isoniazid in South African tuberculosis patients. Br J Clin Pharmacol 72:51–62. doi:10.1111/j.1365-2125.2011.03940.x. 21320152 PMC3141186

[11] Wilkins JJ, Savic RM, Karlsson MO, Langdon G, McIlleron H, Pillai G, Smith PJ, Simonsson US 2008 Population pharmacokinetics of rifampin in pulmonary tuberculosis patients, including a semimechanistic model to describe variable absorption. Antimicrob Agents Chemother 52:2138–2148. doi:10.1128/AAC.00461-07. 18391026 PMC2415769

[12] Evans WE, McLeod HL 2003 Pharmacogenomics: drug disposition, drug targets, and side effects. N Engl J Med 348:538–549. doi:10.1056/NEJMra020526. 12571262

[13] Evans WE, Relling MV 2004 Moving towards individualized medicine with pharmacogenomics. Nature 429:464–468. doi:10.1038/nature02626. 15164072

[14] McIlleron H, Abdel-Rahman S, Dave JA, Blockman M, Owen A 2015 Special populations and pharmacogenetic issues in tuberculosis drug development and clinical research. J Infect Dis 211(Suppl 3):S115–S125. doi:10.1093/infdis/jiu600. 26009615 PMC4551115

[15] Tishkoff SA, Reed FA, Friedlaender FR, Ehret C, Ranciaro A, Froment A, Hirbo JB, Awomoyi AA, Bodo JM, Doumbo O, Ibrahim M, Juma AT, Kotze MJ, Lema G, Moore JH, Mortensen H, Nyambo TB, Omar SA, Powell K, Pretorius GS, Smith MW, Thera MA, Wambebe C, Weber JL, Williams SM 2009 The genetic structure and history of Africans and African Americans. Science 324:1035–1044. doi:10.1126/science.1172257. 19407144 PMC2947357

[16] Kim RB 2003 Organic anion-transporting polypeptide (OATP) transporter family and drug disposition. Eur J Clin Invest 33(Suppl 2):1–5. doi:10.1046/j.1365-2362.33.s2.5.x. 14641549

[17] Tirona RG, Leake BF, Wolkoff AW, Kim RB 2003 Human organic anion transporting polypeptide-C (SLC21A6) is a major determinant of rifampin-mediated pregnane X receptor activation. J Pharmacol Exp Ther 304:223–228. doi:10.1124/jpet.102.043026. 12490595

[18] Chigutsa E, Visser ME, Swart EC, Denti P, Pushpakom S, Egan D, Holford NH, Smith PJ, Maartens G, Owen A, McIlleron H 2011 The SLCO1B1 rs4149032 polymorphism is highly prevalent in South Africans and is associated with reduced rifampin concentrations: dosing implications. Antimicrob Agents Chemother 55:4122–4127. doi:10.1128/AAC.01833-10. 21709081 PMC3165308

[19] Weiner M, Peloquin C, Burman W, Luo CC, Engle M, Prihoda TJ, Mac Kenzie WR, Bliven-Sizemore E, Johnson JL, Vernon A 2010 Effects of tuberculosis, race, and human gene SLCO1B1 polymorphisms on rifampin concentrations. Antimicrob Agents Chemother 54:4192–4200. doi:10.1128/AAC.00353-10. 20660695 PMC2944564

[20] Gengiah TN, Botha JH, Soowamber D, Naidoo K, Abdool Karim SS 2014 Low rifampicin concentrations in tuberculosis patients with HIV infection. J Infect Dev Ctries 18:987–993. doi:10.3855/jidc.4696. 25116663

[21] Ramesh K, Hemanth Kumar AK, Kannan T, Vijayalakshmi R, Sudha V, Manohar Nesakumar S, Bharathiraja T, Lavanya J, Swaminathan S, Ramachandran G 2016 SLCO1B1 gene polymorphisms do not influence plasma rifampicin concentrations in a South Indian population. Int J Tuberc Lung Dis 20:1231–1235. doi:10.5588/ijtld.15.1007. 27510251

[22] Nakajima A, Fukami T, Kobayashi Y, Watanabe A, Nakajima M, Yokoi T 2011 Human arylacetamide deacetylase is responsible for deacetylation of rifamycins: rifampicin, rifabutin, and rifapentine. Biochem Pharmacol 82:1747–1756. doi:10.1016/j.bcp.2011.08.003. 21856291

[23] Shimizu M, Fukami T, Kobayashi Y, Takamiya M, Aoki Y, Nakajima M, Yokoi T 2012 A novel polymorphic allele of human arylacetamide deacetylase leads to decreased enzyme activity. Drug Metab Dispos 40:1183–1190. doi:10.1124/dmd.112.044883. 22415931

[24] van Oosterhout JJ, Dzinjalamala FK, Dimba A, Waterhouse D, Davies G, Zijlstra EE, Molyneux ME, Molyneux EM, Ward S 2015 Pharmacokinetics of antituberculosis drugs in HIV-positive and HIV-negative adults in Malawi. Antimicrob Agents Chemother 59:6175–6180. doi:10.1128/AAC.01193-15. 26248378 PMC4576091

[25] Mitchison DA, Davies GR 2008 Assessment of the efficacy of new anti-tuberculosis drugs. Open Infect Dis J 2:59–76. doi:10.2174/1874279300802010059. 23814629 PMC3694317

[26] Pasipanodya JG, McIlleron H, Burger A, Wash PA, Smith P, Gumbo T 2013 Serum drug concentrations predictive of pulmonary tuberculosis outcomes. J Infect Dis 208:1464–1473. doi:10.1093/infdis/jit352. 23901086 PMC3789573

[27] Carr DF, O'Meara H, Jorgensen AL, Campbell J, Hobbs M, McCann G, van Staa T, Pirmohamed M 2013 SLCO1B1 genetic variant associated with statin-induced myopathy: a proof of concept study using the clinical research practice database. Clin Pharm Ther 94:695–701. doi:10.1038/clpt.2013.161. PMC3831180 23942138

[28] Lubomirov R, di Iulio J, Fayet A, Colombo S, Martinez R, Marzolini C, Furrer H, Vernazza P, Calmy A, Cavassini M, Ledergerber B, Rentsch K, Descombes P, Buclin T, Decosterd LA, Csajka C, Telenti A; Swiss HIV Cohort Study. 2010 ADME pharmacogenetics: investigation of the pharmacokinetics of the antiretroviral agent lopinavir coformulated with ritonavir. Pharmacogenet Genomics 20:217–230. doi:10.1097/FPC.0b013e328336eee4. 20139798

[29] Schuetz EG, Schinkel AH, Relling MV, Schuetz JD 1996 P-glycoprotein: a major determinant of rifampicin-inducible expression of cytochrome P4503A in mice and humans. Proc Natl Acad Sci US 93:4001–4005. doi:10.1073/pnas.93.9.4001. PMC39475 8633005

[30] Goodwin B, Hodgeson E, Liddle C 1999 The orphan human pregnane X preceptor mediates the transcriptional activation of CYP3A4 by rifampicin through a distal enhancer module. Mol Pharmacol 56:1329–1339. 10570062 10.1124/mol.56.6.1329

[31] Choudhri SH, Hawken M, Gathua S, Minyiri GO, Watkins W, Sahai J, Sitar DS, Aoki FY, Long R 1997 Pharmacokinetics of antimycobacterial drugs in patients with tuberculosis, AIDS, and diarrhea. Clin Infect Dis 25:104–111. doi:10.1086/514513. 9243044

[32] McIlleron H, Wash P, Burger A, Norman J, Folb PI, Smith P 2006 Determinants of rifampin, isoniazid, pyrazinamide, and ethambutol pharmacokinetics in a cohort of tuberculosis patients. Antimicrob Agents Chemother 50:1170–1177. doi:10.1128/AAC.50.4.1170-1177.2006. 16569826 PMC1426981

[33] Tappero JW, Bradford WZ, Agerton TB, Hopewell P, Reingold AL, Lockman S, Oyewo A, Talbot EA, Kenyon TA, Moeti TL, Moffat HJ, Peloquin CA 2005 Serum concentrations of antimycobacterial drugs in patients with pulmonary tuberculosis in Botswana. Clin Infect Dis 41:461–469. doi:10.1086/431984. 16028152

[34] van Crevel R, Alisjahbana B, de Lange WC, Borst F, Danusantoso H, van der Meer JW, Burger D, Nelwan RH 2002 Low plasma concentrations of rifampicin in tuberculosis patients in Indonesia. Int J Tuberc Lung Dis 6:497–502. 12068982 10.5588/09640569513002

[35] Chang KC, Leung CC, Yew WW, Chan SL, Tam CM 2006 Dosing schedules of 6-month regimens and relapse for pulmonary tuberculosis. Am J Respir Crit Care Med 174:1153–1158. doi:10.1164/rccm.200605-637OC. 16908866

[36] Li J, Munsiff SS, Driver CR, Sackoff J 2005 Relapse and acquired rifampin resistance in HIV-infected patients with tuberculosis treated with rifampin- or rifabutin-based regimens in New York City, 1997-2000. Clin Infect Dis 41:83–91. doi:10.1086/430377. 15937767

[37] Weiner M, Benator D, Burman W, Peloquin CA, Khan A, Vernon A, Jones B, Silva-Trigo C, Zhao Z, Hodge T; Tuberculosis Trials Consortium. 2005 Association between acquired rifamycin resistance and the pharmacokinetics of rifabutin and isoniazid among patients with HIV and tuberculosis. Clin Infect Dis 40:1481–1491. doi:10.1086/429321. 15844071

[38] Boeree MJ, Diacon AH, Dawson R, Narunsky K, du Bois J, Venter A, Phillips PP, Gillespie SH, McHugh TD, Hoelscher M, Heinrich N, Rehal S, van Soolingen D, van Ingen J, Magis-Escurra C, Burger D, Plemper van Balen G, Aarnoutse RE; PanACEA Consortium. 2015 A dose-ranging trial to optimize the dose of rifampin in the treatment of tuberculosis. Am J Respir Crit Care Med 191:1058–1065. doi:10.1164/rccm.201407-1264OC. 25654354

[39] Oken MM, Creech RH, Tormey DC, Horton J, Davis TE, McFadden ET, Carbone PP 1982 Toxicity and response criteria of the Eastern Cooperative Oncology Group. Am J Clin Oncol 5:649–655. doi:10.1097/00000421-198212000-00014. 7165009

[40] Sloan DJ, Mwandumba HC, Garton NJ, Khoo SH, Butterworth AE, Allain TJ, Heyderman RS, Corbett EL, Barer MR, Davies GR 2015 Pharmacodynamic modeling of bacillary elimination rates and detection of bacterial lipid bodies in sputum to predict and understand outcomes in treatment of pulmonary tuberculosis. Clin Infect Dis 61:1–8. doi:10.1093/cid/civ195. 25778753 PMC4463005

[41] Malawi Ministry of Health. 2007 National tuberculosis control programme manual, 6th ed Ministry of Health, Lilongwe, Malawi.

[42] Mlotha R, Waterhouse D, Dzinjalamala F, Ardrey A, Molyneux E, Davies GR, Ward S 2015 Pharmacokinetics of anti-TB drugs in Malawian children: reconsidering the role of ethambutol. J Antimicrob Chemother 70:1798–1803. doi:10.1093/jac/dkv039. 25759035 PMC4498297

